# SMCHD1 regulates a limited set of gene clusters on autosomal chromosomes

**DOI:** 10.1186/s13395-017-0129-7

**Published:** 2017-06-06

**Authors:** Amanda G. Mason, Roderick C. Slieker, Judit Balog, Richard J. L. F. Lemmers, Chao-Jen Wong, Zizhen Yao, Jong-Won Lim, Galina N. Filippova, Enrico Ne, Rabi Tawil, Bas T. Heijmans, Stephen J. Tapscott, Silvère M. van der Maarel

**Affiliations:** 1 Human Genetics, Leiden University Medical Center, Leiden, The Netherlands; 2 Molecular Epidemiology, Leiden University Medical Center, Leiden, The Netherlands; 3 Division of Human Biology, Fred Hutchinson Cancer Research Center, Seattle, WA USA; 4 Neuromuscular Disease Unit, Department of Neurology, University of Rochester Medical Center, Rochester, NY USA; 5 Netherlands Consortium for Healthy Aging, Leiden, The Netherlands

**Keywords:** SMCHD1, FSHD, Chromatin, Methylation

## Abstract

**Background:**

Facioscapulohumeral muscular dystrophy (FSHD) is in most cases caused by a contraction of the D4Z4 macrosatellite repeat on chromosome 4 (FSHD1) or by mutations in the *SMCHD1* or *DNMT3B* gene (FSHD2). Both situations result in the incomplete epigenetic repression of the D4Z4-encoded retrogene *DUX4* in somatic cells, leading to the aberrant expression of DUX4 in the skeletal muscle. In mice, Smchd1 regulates chromatin repression at different loci, having a role in CpG methylation establishment and/or maintenance.

**Methods:**

To investigate the global effects of harboring heterozygous *SMCHD1* mutations on DNA methylation in humans, we combined 450k methylation analysis on mononuclear monocytes from female heterozygous *SMCHD1* mutation carriers and unaffected controls with reduced representation bisulfite sequencing (RRBS) on FSHD2 and control myoblast cell lines. Candidate loci were then evaluated for SMCHD1 binding using ChIP-qPCR and expression was evaluated using RT-qPCR.

**Results:**

We identified a limited number of clustered autosomal loci with CpG hypomethylation in *SMCHD1* mutation carriers: the protocadherin (*PCDH*) cluster on chromosome 5, the transfer RNA (tRNA) and 5S rRNA clusters on chromosome 1, the *HOXB* and *HOXD* clusters on chromosomes 17 and 2, respectively, and the D4Z4 repeats on chromosomes 4 and 10. Furthermore, minor increases in RNA expression were seen in FSHD2 myoblasts for some of the *PCDH*β cluster isoforms, tRNA isoforms, and a *HOXB* isoform in comparison to controls, in addition to the previously reported effects on *DUX4* expression. SMCHD1 was bound at DNAseI hypersensitivity sites known to regulate the *PCDH*β cluster and at the chromosome 1 tRNA cluster, with decreased binding in *SMCHD1* mutation carriers at the *PCDH*β cluster sites.

**Conclusions:**

Our study is the first to investigate the global methylation effects in humans resulting from heterozygous mutations in *SMCHD1*. Our results suggest that SMCHD1 acts as a repressor on a limited set of autosomal gene clusters, as an observed reduction in methylation associates with a loss of SMCHD1 binding and increased expression for some of the loci.

**Electronic supplementary material:**

The online version of this article (doi:10.1186/s13395-017-0129-7) contains supplementary material, which is available to authorized users.

## Background

The chromatin modifier *SMCHD1* (structural maintenance of chromosomes flexible hinge domain containing 1) is an atypical member of the *SMC* (structural maintenance of chromosomes) gene superfamily containing both a hinge and ATPase domain. Traditional SMC proteins form heterodimers and together with other non-SMC proteins create the cohesin and condensin complexes known to regulate chromatin repression and RNA-directed DNA methylation in many species, with specific involvement in sister chromatid cohesion, gene regulation, DNA repair, and chromosome architecture ([1–4] and reviewed in [5]). In contrast, *Smchd1* forms homodimers [6, 7] and was initially identified in a mouse mutagenesis screen for modifiers of variegated expression of a multi-copy transgene, where it was found to be a suppressor of variegation [8]. The initial and follow-up studies in mice have since showed that Smchd1 is necessary for the establishment and/or maintenance of methylation of a subset of CpG islands associated with X-inactivation and some autosomal loci that undergo monoallelic expression, such as imprinted genes and the protocadherin (*Pcdh*) gene cluster [9–12].

The majority of facioscapulohumeral muscular dystrophy (FSHD)2 cases have been attributed to heterozygous mutations in *SMCHD1* [13, 14]. FSHD is a relatively common muscular dystrophy with an estimated prevalence of 1:8,500–1:20,000 [15, 16], initially characterized by the progressive atrophy of facial and upper extremity muscles, but with disease progression, other muscles may become affected. FSHD is caused by the incomplete epigenetic repression of the double homeobox 4 (*DUX4*) retrogene, one copy of which is located in each unit of the D4Z4 macrosatellite repeat array on chromosome 4, most often either by D4Z4 repeat array contraction (FSHD1) or by mutations in the *SMCHD1* gene (FSHD2), in conjunction with a permissive chromosome haplotype [17, 18]. *DUX4* encodes for a double homeobox transcription factor that is most abundantly expressed in the luminal cells of the testis and repressed in somatic cells [17, 19]. The polymorphic D4Z4 array consists of 8–100 D4Z4 units of 3.3 kb in unaffected individuals, while patients with FSHD1, the more common form of FSHD, have a shortened array of 1–10 units (reviewed [20]). The *DUX4* retrogene makes use of sequences immediately distal to the D4Z4 repeat array that contain a polymorphic polyadenylation signal (PAS), which are essential for the production of a stable DUX4 messenger RNA (mRNA) in somatic cells. *DUX4*-PAS-containing chromosome 4s, referred to as 4qA, account for approximately half of the population’s chromosome 4s and are required for FSHD susceptibility. In contradistinction, contractions on chromosomes that do not contain a DUX4-PAS (4qB chromosomes), or chromosome 10s that contain a highly homologous repeat array, fail to produce stable DUX4 and hence do not cause FSHD [21–24]. Thus, FSHD2, in most cases, is caused by the digenic inheritance of an *SMCHD1* mutation in combination with a *DUX4*-PAS-containing chromosome 4qA. Recently, we showed that in a minority of FSHD2 patients who do not have an *SMCHD1* mutation, the digenic inheritance of a mutation in the DNA methyltransferase 3B (*DNMT3B*) gene and a *DUX4*-PAS-containing chromosome 4qA can also derepress *DUX4* in myocytes [25]. Additionally, some FSHD cases remain unexplained, as the causative mutations have not yet been identified.

In FSHD patients, *DUX4* repression is incomplete as evidenced by a partial loss of D4Z4 CpG methylation and repressive histone modifications in somatic cells, indicative of a partial relaxation of the chromatin structure at the D4Z4 repeat array [26–28]. SMCHD1 plays a largely uncharacterized role in the somatic repression of *DUX4* through direct binding at the D4Z4 repeat array in somatic cells [13]. Mutations in *SMCHD1*, in FSHD2 families, lead to a decrease in SMCHD1 protein levels and reduced binding of SMCHD1 to the D4Z4 repeat array, resulting in D4Z4 hypomethylation and incomplete repression of *DUX4* in the skeletal muscle [13]. Additionally, there is a direct effect of *SMCHD1* mutations influencing the methylation level at the D4Z4 repeat in FSHD1 individuals, leading to an exacerbation of D4Z4 hypomethylation and symptoms as well as increased DUX4 mRNA expression [14, 29]. However, within FSHD2 families, there are *SMCHD1* mutation carriers that do not develop FSHD due to the absence of a permissive *DUX4*-PAS chromosome 4qA in their genome, and they do not present with any other overt symptoms [21].

The absence of an explicit disease phenotype in non-FSHD presenting *SMCHD1* mutation carriers prompted us to investigate the genome-wide effects of harboring a heterozygous *SMCHD1* mutation. Additionally, studying SMCHD1’s global genomic role could give insight to potential consequences in FSHD and for mutation carriers. Thus, we explored the effects of heterozygous *SMCHD1* mutations on global DNA methylation. We discovered a loss of methylation at certain autosomal gene clusters by performing a 450k array methylation analysis comparing 24 *SMCHD1* mutation carriers and 23 controls not carrying an *SMCHD1* mutation as well as reduced representation bisulfite sequencing (RRBS) on two *SMCHD1* mutation carriers and two controls. SMCHD1 binding at regulatory sites of some of these clusters was then confirmed, and we found expression differences of certain cluster isoforms between control and FSHD2 individuals.

## Methods

A summary of all patient materials used in these studies is listed in Additional file 1: Table S1.

### 450k

Genomic DNA (500 ng) was bisulfite converted using the EZ DNA Methylation kit (Zymo Research, cat# D5020). Converted DNA was hybridized onto the Illumina Infinium 450k array according to the manufacturer’s protocols. Signal intensities were measured using an Illumina iScan BeadChip scanner. Data was normalized using functional normalization in the Bioconductor package *minfi* using five principal components [30, 31]. Probes that ambiguously mapped or had a high detection *P* value (>0.01), low bead count (<3 beads), and a low success rate (missing in >95% of the samples) were set to missing. All CpG probes were analyzed independently.

### RRBS procedure and analysis

RRBS: Sample library preparation (EpiQuest) and data analysis for RRBS were performed by service provider (Zymo Research) as described [32, 33]. EpiQuest libraries were prepared from 200 to 500 ng of human gDNA. The DNA was digested with 60 units of TaqI and 30 units of MspI sequentially. Size-selected TaqI-MspI fragments (40–120 and 120–350 bp) were filled-in and 30-terminal-A extended, extracted with Zymo Research DNA Clean and Concentrator kit (cat# D4003). Ligation to pre-annealed adaptors containing 5-methyl-cytosine instead of cytosine (Illumina) was performed using the Illumina DNA preparation kit and protocol. Purified, adaptor-ligated fragments were bisulfite treated using the EZ DNA Methylation-Direct Kit (Zymo Research, cat# D5020). Preparative-scale PCR was performed and DNA Clean and Concentrator-purified PCR products were subjected to a final size selection on a 4% NuSieve 3:1 agarose gel. SYBR green-stained gel slices containing adaptor-ligated fragments of 130–210 or 210–460 bp in size were excised. Library material was recovered from the gel (Zymoclean Gel DNA Recovery Kit, cat# D4001) and sequenced on an Illumina GAIIx genome analyzer. Sequence reads from bisulfite-treated EpiQuest libraries were identified using standard Illumina base-calling software.

Alignment and methylation calls: Reads were mapped to human (hg19) genome using BSMAP (version 2.74), with the option “-q 20 –AAGATCGGAAGAGC” to trim adaptor sequences and low-quality base calls [34]. The numbers of unconverted and converted cytosine (C) reads covering the loci for each sample were extracted using python scripts methyratio.py in the BSMAP package.

CpG statistics: It has been shown that methylation intensity among close by CpG loci are highly correlated. Hence, instead of considering methylation calls on each CpG locus, CpG blocks were composed by aggregating CpG loci within 50 bps from each other. The total coverage for a CpG block is the sum of unconverted and converted C reads for all containing CpG loci, and the methylation ratio is computed as the ratio of the sum of unconverted C reads covering all containing CpG loci against the total coverage. The *P* value significance was computed by the *chi*-*square* test using the number of unconverted C reads and total coverage for each sample. Differential methylation calls for a CpG block (DMB) were determined by the *P* value and the difference of methylation ratios between the samples from different biological conditions.

To identify larger regions of differential methylation, the CpG blocks within 5000 bps up/downstream were merged. Some uninteresting CpG blocks were excluded; these included CpG blocks with less than ten total read across samples and those having greater than one mismatch across samples. A differentially methylated region (DMR) was called if a region contained at least ten DMBs with corresponding *P* value <0.01 and a difference in methylation ratio greater than 0.1.

### Bisulfite PCR analysis

Bisulfite treatment of 500 ng DNA was done using the Zymo EZ DNA Methylation-Lightning Kit (Zymo Research, cat #D5031) with their standard protocol. Two microliters of bisulfite-treated DNA was amplified using bisulfite-specific PCR primers. PCR products were purified using the Nucleospin Gel and PCR clean-up kit (Macherey-Nagel, cat# 740609). Purified products were either directly sequenced using Sanger sequencing and chromatograph traces analyzed with the ESME analysis software [35] or first cloned into the TOPO-TA vector (ThermoFisher, cat# 450641) where single colonies were miniprepped and sequenced using sanger sequencing.

### Culturing of primary human myoblast cells

Human primary myoblast cell lines originated from the University of Rochester biorepository (https://www.urmc.rochester.edu/fields-center.aspx). Myoblast cells were grown in DMEM/F-10 medium (Gibco, #31550) with 20% heat-inactivated fetal bovine serum (Gibco, FBS #10270), 1% penicillin/streptomycin (Gibco, #15140), 10 ng/mL rhFGF (Promega, #G5071), and 1 μM dexamethasone (Sigma-Aldrich, #D2915) at 37 °C with 5% CO_2_. Myoblast were fused at 80–90% confluency by culturing cells in DMEM GlutaMAX (Gibco, #31966), with 2% KnockOut serum (Gibco, #10828) for 48 h.

### Viral transduction of primary myoblast

Primary myoblast cells were transduced at 50% confluency with lentiviral particles, 3 ng/cm^2^ (viral titter determined by HIV-1 p24 Antigen ELISA 2.0 kit (Zeptometrix Corporation)), containing either control (shTurboGFP (SHC004) and shLuciferase (SHC007)) or SMCHD1 short hairpin RNAs (shRNAs) (TRCN0000253778 and TRCN0000253776) obtained from the Mission shRNA library (MISSION shRNA library, Sigma-Aldrich). At 24 h post transduction, the virus was washed off followed by puromycin selection 48 h post transduction by the addition of media containing 1 mg/ml puromycin (Sigma-Aldrich, P8833).

### Chromatin immunoprecipitation

SMCHD1 ChIP was carried out as previously described [36]. In brief, cells were cross-linked for 10 min using 1% formaldehyde followed by quenching using glycine at an end concentration of 125 mM for 5 min. Cross-linked cells were then lysed and chromatin shearing was carried out using a Bioruptor UCD-20 (four times 10 min, 15 s on/off cycles, max output); 30 μg of chromatin was precleared followed by overnight incubation at 4 °C with either 5 μg/rxn anti-SMCHD1 (Abcam, ab31865) or 5 μg/rxn of rabbit IgG (Millipore, PP64-K) to control for non-specific binding. All experiments were carried out at least two independent times for all samples. Relative fold enrichment of SMCHD1 at a locus was calculated relative to IgG-binding background levels and normalized for genomic DNA input using quantitative qPCR CT values. Lastly, all values are depicted as the ΔΔCT, where SMCHD1 levels are shown normalized to IgG levels set to 1; error bars represent the SEM of biological replicates. ChIP primers are listed in Additional file 2: Table S2.

### RNA isolation, cDNA generation, and RT-qPCR

Myoblast and myotube samples for RNA isolation were harvested by the direct addition of QIAzol lysis reagent (Qiagen, #79306). RNA was then isolated using miRNeasy Mini Kit (Qiagen, #217004) including DNase treatment according to the manufacturer’s instructions. Complementary DNA (cDNA) generation was done using RevertAid H Minus First strand cDNA Synthesis Kit (Thermo Fischer Scientific Inc, #K1632) with 2 μg RNA template and poly-dT primers for all studies except transfer RNA (tRNA) and 5S ribosomal RNA (rRNA) studies where random hexamers were used. Gene expression was quantified using qPCR in duplicate using SYBR green master mix (170-8886, BIO-RAD). Gene-specific primers were used and are listed in Additional file 2: Table S2; *GUS1* was used as the housekeeping gene.

### Statistics

Statistics for comparing between the control group (control individuals or control shRNAs) and the group of interest (FSHD1, FSHD2, or SMCHD1 shRNAs) was done using a *t* test with Bonferroni correction. Significance is denoted as (*) and was determined by Bonferroni adjusted *P* values <0.05. Statistical methods for the 450k array and RRBS are listed in their appropriate methods section.

## Results

### 450k methylation analysis identifies hypomethylation of autosomal gene clusters in *SMCHD1* mutation carriers

**Fig. 1:**
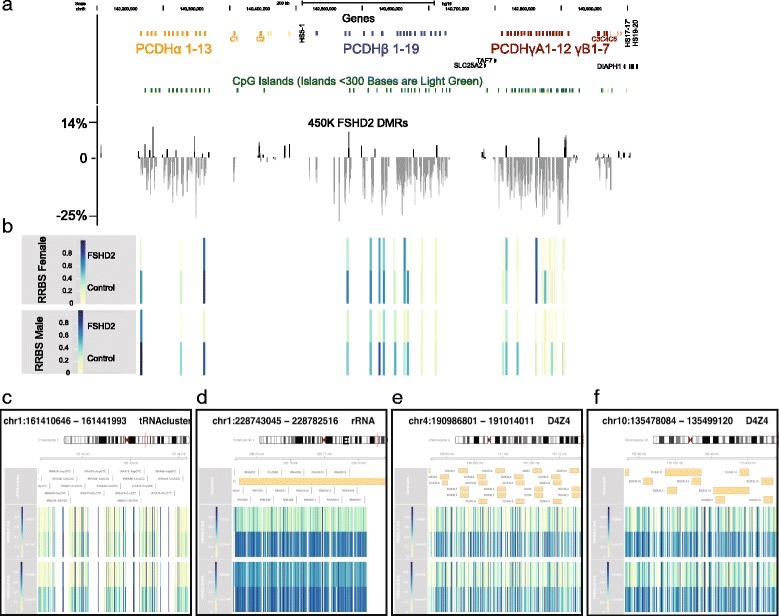
Hypomethylation of the *PCDH* cluster. **a** The *top track* depicts the location of each clustered gene as annotated by RefSeq and denotes the location of the hypersensitivity sites HS5-1 just distal of the *PCDH*α cluster and HS17-17′ and HS19-20 just distal of the *PCDH*γ cluster. The *second track* depicts all annotated CpG islands in the region, with CpG islands less than 300 bases being depicted in *light green*. The *third track* of the figure depicts the relative methylation difference in 24 *SMCHD1* mutation carrying individuals in comparison to 23 controls at each recorded CpG in the 450k methylation analysis in the region of the clustered protocadherins on chromosome 5. The methylation value in controls was set to the baseline of 0 for each CpG. **b** Methylation heat maps from the RRBS analysis across the clustered protocadherin locus representing the relative methylation between control and FSHD2 individuals. The color scale is from yellow, no/low number of methylated CpGs at the locus to dark blue, high number of methylated CpGs. **c**–**f** Figures depict heat maps of relative methylation levels found by RRBS in FSHD2 and control individuals at the locations within (**c**) the tRNA cluster on chromosome 1, (**d**) the 5S rRNA cluster on chromosome 1, (**e**) the D4Z4 macrosatellite repeat on chromosome 4, and (**f**) the D4Z4 macrosatellite repeat on chromosome 10

To explore the genome-wide CpG methylation effects of *SMCHD1* mutations, we compared the methylome in peripheral blood mononuclear cells of 24 female *SMCHD1* mutation with 23 unaffected female controls (of which 15 were from FSHD2 families but lacked the familial *SMCHD1* mutation). The Illumina 450k array was used to profile genome-wide DNA methylation levels in the two groups. We first analyzed and identified individual CpGs across the autosomes that were differentially methylated according to the stringent criteria (*P*_FDR_ < 0.05; delta-methylation >5%; 2043 CpGs) and subsequently combined them into robust differentially methylated regions (DMRs) using the DMRfinder algorithm [37]. This resulted in the identification of 80 DMRs (Additional file 3: Table S3). DMRs were primarily restricted to the proximal promoters (CpG islands and shores, collectively 60% of all DMR loci) and intergenic CpG islands (13% of DMRs). Interestingly, the majority of autosomal DMRs identified were located within or adjacent to gene clusters, e.g., the *HOXB* and *HOXD* gene clusters on chromosomes 17 and 2, respectively, the clustered *TBX3* and *TBX5* genes on chromosome 12, the clustered *ZIC1* and *ZIC4* genes on chromosome 3, and the *ZNF* cluster on chromosome 7. The most striking finding was that the majority, 48 of the 80 DMRs, mapped to locations within the protocadherin (*PCDH*) gene cluster on chromosome 5. Detailed examination of this cluster revealed that almost all isoforms of the *PCDH* gene cluster have hypomethylated CpGs in their promoter and/or gene bodies (51 of the 57 isoforms within the cluster) in *SMCHD1* mutation carriers ranging from a 5–20% reduction in DNA methylation (Fig. 1a). We find that the *PCDH* cluster is the most effected hypomethylated autosomal gene cluster in *SMCHD1* mutation carriers. This is consistent with previous studies in homozygous mutant Smchd1 mice where the *Pcdh* cluster was shown to be regulated by SMCHD1 activity in neuronal cells and during development [11, 12, 38]. In addition, SMCHD1 ChIP-seq studies in mouse neural stem cells have identified SMCHD1 binding sites at the *Pcdh* cluster, the *Hoxb* and *Hoxd* clusters, and the clustered *Tbx5* and *Tbx3* genes [38].

### Genome-wide methylation analysis of *SMCHD1* mutation carriers reveals SMCHD1 regulation at repetitive gene clusters

The 450k array is limited in its coverage of repetitive regions, which includes a number of gene clusters in the human genome. Thus, to fully capture the extent of *PCDH* cluster and D4Z4 hypomethylation, validate the 450k array in an FSHD relevant cell type, and to explore additional repetitive regions not represented on the 450k array where methylation may be affected in FSHD2 individuals, RRBS was carried out on myoblasts derived from four individuals. Myoblast cells from two FSHD2 individuals (one female and one male) and two unaffected controls (one female and one male) were used for this analysis. CpGs within 50 base pairs (CpG blocks) with similar methylation values were grouped to identify differentially methylated blocks (DMBs) by using chi-square test *P* value <0.01 and difference in methylation ratios >0.1 between FSHD2 and control samples. Differential methylation analysis was done separately for the male and female samples due to large biological differences between the two sexes. After DMBs were identified, clustering of DMBs within 5000 bps was done to identify DMRs, defined as a region containing 10 or more DMBs. We found that differentially hypomethylated autosomal DMBs were relatively equally distributed between the male and female samples.

On the autosomal chromosomes, we identified five hypomethylated regions with clustered loci in both the female and male FSHD2 sample. Consistent with the findings of the 450k array one of these regions was the *PCDH* gene cluster on chromosome 5 (Fig. 1b). The four other regions were the 5S rRNA cluster on chromosome 1, the tRNA cluster on chromosome 1, and, as expected from previous studies, the two D4Z4 repeat arrays on chromosomes 4 and 10, which provided further confidence in the dataset (Additional file 4: Table S4) (Fig. 1c–f). There were also two regions that were hypomethylated only in the male FSHD2 sample, the *TCEB3C* cluster on chromosome 18 and the *ZAV* macrosatellite repeat on chromosome 9. We examined these regions in additional control and FSHD2 individuals using bisulfite sequencing experiments and found that the *TCEB3C* cluster was highly methylated in all individuals, while the *ZAV* macrosatellite repeat appears to be more hypomethylated in FSHD2 individuals, but controls show a high degree of variation (Additional file 5: Figure S1).

### SMCHD1 binds the CCR regulatory region of *PCDH* cluster in primary myoblast cells

The *PCDH* cluster on chromosome 5 was consistently the most significantly hypomethylated region in FSHD2 cells identified using both methodologies. Additionally, this region had been identified in mouse studies [38, 39] and a cell line study [40] as being Smchd1/SMCHD1 regulated. Moreover, mutations in *SMCHD1* alter the epigenetic architecture of the entire *PCDH* cluster region [38]. Thus, these studies in conjunction with our findings suggest that the PCDH cluster is under regulation by SMCHD1 in somatic cells. To determine whether SMCHD1 might be affecting cluster regulation through binding at known regulatory hypersensitivity sites (HS) in muscle cells, we took a chromatin immunoprecipitation followed by quantitative PCR (ChIP-qPCR) approach. We examined the PCDHα regulatory site HS5-1 and the PCDHβ cluster control region (CCR) HS sites HS17-17′ and HS19-20 [41]. The CCR consists of six HS sites, HS16, 17, 17′, 18, 19, and 20, but due to proximity, HS17-17′and HS19-20 are each referred to as one unit.

**Fig. 2:**
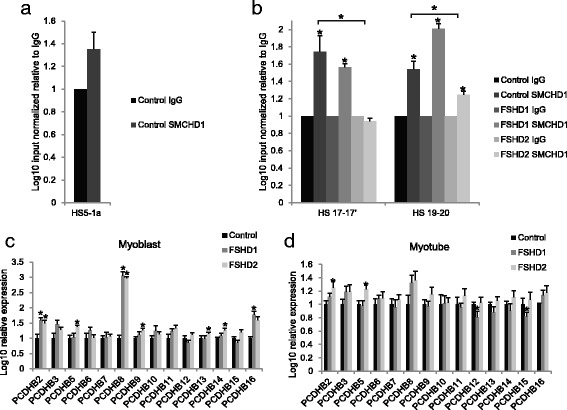
SMCHD1 binding to the PCDH cluster control region and transcriptional expression of the *PCDH*β cluster. qPCR analysis on SMCHD1 ChIPed DNA for HS regions known to control the expression of PCDHα, (**a**) HS5-1 and PCDHβ, (**b**) HS17-17′and HS19-20, and cluster isoforms. Results represent the log10 input normalized binding enrichment relative to IgG, which has been arbitrarily set to 1. *Asterisks directly over bars* represent a significant increase in binding relative to IgG; *asterisks* on a line indicate a significant difference in binding between cell types. *Bonferroni adjusted *P* < 0.05, *t* test; *n* = 4 for controls, *n* = 2 for FSHD1, *n* = 5 for FSHD2; *error bars* = SEM. RNA expression analysis of *PCDH*β cluster isoform members in primary control, FSHD1, and FSHD2 (**c**) myoblast and (**d**) myotube cells. Results represent qRT-PCR analysis of the indicated gene after normalization to the internal control gene *GUS1*. For each gene, the log10 value of expression is given relative to control individuals where this value was arbitrarily set to 1. *Bonferroni adjusted *P* < 0.05, *t* test; *n* = 4 for controls, *n* = 10 for FSHD1, *n* = 7 for FSHD2; *error bars* = SEM

SMCHD1 binding was first examined using primary myoblast cell lines from four unaffected individuals. Significant SMCHD1 binding over IgG levels was identified at the CCR HS sites HS17-17′ and HS19-20 but not at HS5-1 (Fig. 2a, b). We then explored SMCHD1 binding at the two CCR HS sites in myoblast cells from two FSHD1 individuals and five FSHD2 individuals harboring heterozygous *SMCHD1* mutations. Significant SMCHD1 binding over IgG was identified at the HS sites HS17-17′ and HS19-20 in FSHD1 cells and at HS19-20 in FSHD2 cells, while binding was significantly reduced in the FSHD2 cells at both sites compared to cells from the unaffected individuals (Fig. 2b).

### Loss of SMCHD1 binding at *PCDH* CCR HS sites in myoblasts results in *PCDH*β transcriptional changes

As our ChIP-qPCR studies indicated SMCHD1 binding at CCR HS sites, and a previous study comparing expression in FSHD1 and FSHD2 muscle cells found that *PCHDB2* was more highly expressed in FSHD2 cells [42], we next determined expression of *PCDH* cluster genes in FSHD. The PCDHα cluster has very low expression in muscle cells; thus, we focused on the expression levels of the *PCDH*β and *PCDH*γ cluster isoforms comparing the expression in cultured muscle cells from four unaffected, ten FSHD1, and seven FSHD2 individuals. In myoblasts, the expression level of 14 *PCDH*β isoforms was examined and six (*PCDH* β*2*, β*5*, β*8*, β*9*, β*13*, and β*14*) had significantly increased expression in FSHD2 myoblast cells (Fig. 2c), while in myotube cells, *PCDH* β*2* and β*5* were significantly increased (Fig. 2d). In FSHD1 myoblast cell lines, we surprisingly also found an increase in the expression of three *PCDH*β isoforms (*PCDH* β*2*, β*8*, and β*16*) (Fig. 2c), while FSHD1 myotube cells showed significant reductions in *PCDH* β*12* and β*15* (Fig. 2d) (*Bonferroni adjusted *P* < 0.05). A total of 11 *PCDH*γ isoforms were examined by qPCR. No significant differences were found between controls and FSHD2 myoblast cells, while *PCDH*γ*B7* was significantly decreased in myotube cells (Additional file 6: Figure S2). In FSHD1 myoblast cells, *PCDH*γ*A3* and *PCDH*γ*B7* were increased, while *PCDH*γ*A2* was increased in myotube cells (Additional file 6: Figure S2). This supports the conclusion that the loss of SMCHD1 binding at the CCR HS sites is important for controlling *PCDH*β cluster expression in FSHD2 myoblast cells and to some extent in differentiated myotubes, yet we cannot attribute expression changes in FSHD1 cells to changes in SMCHD1 binding at CCR regulatory sites.

### Reduction in SMCHD1 in individuals carrying two chromosomes that are non-permissive for DUX4 expression

**Fig. 3:**
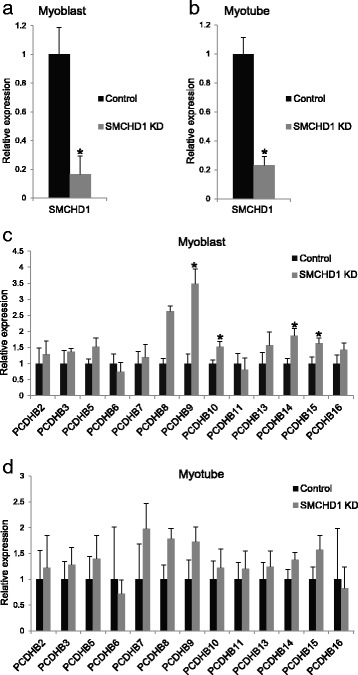
The effects of SMCHD1 KD in non-DUX4 permissive 4qB cells on *PCDH*β cluster expression. The expression level of SMCHD1 after SMCHD1 shRNA KD in comparison with control KD shRNAs targeting luciferase and GFP is shown by RT-qPCR analysis in primary (**a**) myoblast and (**b**) myotube cells. RNA expression analysis was then assessed for *PCDH*β cluster isoform members in primary (**c**) myoblast and (**d**) myotube cells. Results represent qRT-PCR analysis of the indicated gene after normalization to the internal control gene *GUS1*. For each gene, the value of expression in the control shRNA samples was then arbitrarily set to 1. *P < 0.05, *t* test; *n* = 6 (two shRNA constructs for each target on three independent cell lines); *error bars* = SEM

Our results that some *PCDH*β isoforms had significant expression differences in FSHD1 cells in comparison to controls were surprising. However, examination of previously published DUX4 ChIP-seq data [43] showed DUX4 binding at HS16 within the CCR (Additional file 7: Figure S3); thus, DUX4 may also have a transcriptional role at the *PCDH* cluster. FSHD2 cells have mutant SMCHD1 but also express DUX4. Therefore, to determine if SMCHD1 has a direct effect on the locus in the absence of DUX4, we reduced SMCHD1 levels in three myoblast cell lines that contain two non-permissive 4qB chromosomes, and thus do not produce a stable DUX4 transcript. We used two independent SMCHD1 targeting shRNAs to knock down SMCHD1 compared to two control shRNAs (Fig. 3a, b) and verified that there was no DUX4 or DUX4 target expression with SMCHD1 KD (Additional file 8: Figure S4A). In the 4qB SMCHD1 knockdown myoblast cells, despite the short-term nature of the SMCHD1 reduction, there was a significant upregulation of some *PCDH*β isoforms in the absence of DUX4 expression (*PCDH*β*9*, β*10*, β*14*, β*15*) (Fig. 3c), while in the differentiated myotubes, there were no significant differences (Fig. 3d). No genes in the *PCDH*γ cluster were increased following SMCHD1 knockdown in either the myoblasts or the myotubes (Additional file 8: Figure S4B, C). This data shows that loss of SMCHD1, even in a short-term reduction, does affect the expression of many *PCDH*β isoforms independent of gene expression changes at the locus that may be regulated by DUX4 expression in FSHD1 and FSHD2 individuals.

### SMCHD1 binds within the tRNA cluster on chromosome 1

**Fig. 4:**
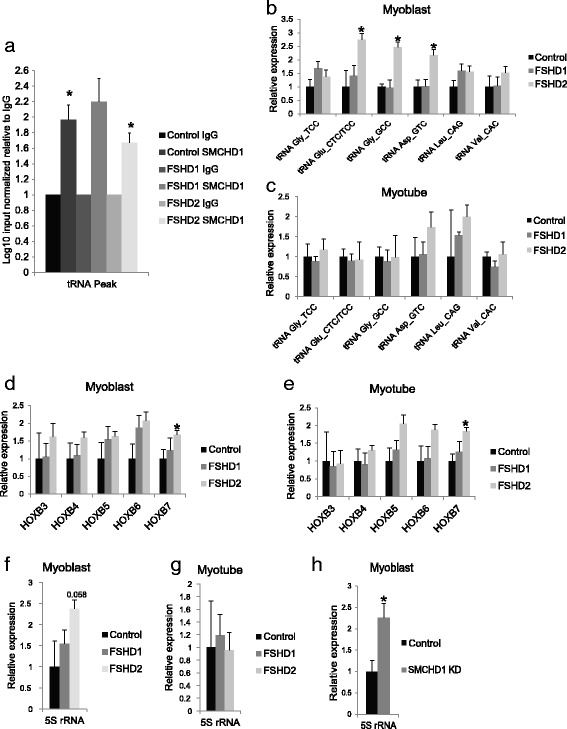
SMCHD1 binding at the tRNA cluster and transcriptional expression of the chromosome tRNA, *HOXB*, and 5S rRNA clusters. **a** qPCR analysis on SMCHD1 ChIPed DNA for the identified SMCHD1 binding site on chromosome 1 within the tRNA cluster using *n* = 4 for control, *n* = 2 for FSHD1, and *n* = 5 for FSHD2 myoblast cell lines. Results represent the log10 input normalized binding enrichment relative to IgG, which has been arbitrarily set to 1. *Asterisks directly over bars* represent a significant increase in binding relative to IgG *Bonferroni adjusted *P* < 0.05, *t* test; *error bars* = SEM. RNA expression analysis on isoforms within the chromosome 1 tRNA cluster and tRNA Val_CAC directly proximal to the cluster repeat in primary (**b**) myoblast and (**c**) myotube cells, the *HOXB* cluster isoform members in primary (**d**) myoblast and (**e**) myotube cells, and the 5S rRNA cluster on chromosome 1 in (**f**) myoblast and (**g**) myotube cells. RNA expression analysis for the 5S rRNA cluster on chromosome 1 for (**h**) myoblasts in SMCHD1 shRNA KD in comparison with control KD shRNAs in non-DUX4 permissive 4qB cells. Results represent qRT-PCR analysis of the indicated gene after normalization to the internal control gene *GUS1*. For each gene, the value of expression in control individuals was then arbitrarily set to 1. *Bonferroni adjusted *P* < 0.05, *t* test; *n* = 4 for controls, *n* = 10 for FSHD1, *n* = 7 for FSHD2, *n* = 6 for non-DUX4 permissive 4qB cells (two shRNA constructs for each target on three independent cell lines); *error bars* = SEM

A recent SMCHD1 ChIP-seq study carried out in HCT116 human colon carcinoma cells identified an SMCHD1 binding peak within the region of the tRNA cluster on chromosome 1 at (chr1:161,423,907-161,423,948) (Nowak A, Armenise C, Rey E, Déjardin J GSE46462). This region is highly repetitive and has been identified as a tandem repeat locus with extensive copy number variation, ranging from 9–43 repeat units [44, 45]. To test whether SMCHD1 was bound to this region in myoblast cells from control, FSHD1, and FSHD2 individuals, we carried out ChIP-qPCR. We found that there was significant binding of SMCHD1 over IgG to the identified peak sequence in control and FSHD2 samples to a similar level as at the D4Z4 repeat (Fig. 4a, Additional file 9: Figure S5); however, no loss of binding was seen in FSHD2 samples compared to controls.

### Expression of tRNAs within the cluster on chromosome 1 are altered in FSHD2 individuals

Using qPCR, we explored whether the reduction in methylation and SMCHD1 binding in FSHD2 individuals at the tRNA cluster on chromosome 1 affected the expression of tRNA isoforms in the region. The region consists of a repeating set of five tRNA isoforms: Gly_GCC, Glu_CTC, Gly_TCC, ASP_GTC, and Leu_CAG. We determined the expression of these tRNAs, as well as that of tRNA Val_CAC found upstream of the repeated tRNAs, in control, FSHD1, and FSHD2 myoblast and myotube cells. We found that expression of tRNAs Gly_GCC, Glu_CTC/TCC (primers do not distinguish), and Asp_GTC had significant upregulation in FSHD2 myoblast cells compared to controls (*Bonferroni adjusted *P* < 0.05) (Fig. 4b). No significant differences were seen in myotube cells (Fig. 4c). This suggests that despite not picking up a binding difference by ChIP-qPCR at the tRNA cluster in FSHD2 samples, reduced SMCHD1 activity is associated with the observed hypomethylation and gene expression differences at the locus.

### Gene expression differences within other hypomethylated clusters in FSHD2 individuals

The TBX3/TBX5, the *HOXB*, and the *HOXD* clusters were also identified as having hypomethylated CpGs using the 450k array. In addition, SMCHD1 binding peaks were identified by ChIP in the mouse *Hoxb*, *Hoxd*, and *Tbx5*/*Tbx3* clusters [38]. Within the *HOXB* cluster, we examined the expression of five cluster members. In myoblast cells of FSHD2 individuals compared to controls, we only found a significant increase in *HOXB7* (*Bonferroni adjusted *P* < 0.05) (Fig. 4d). This increase was also seen in FSHD2 myotubes (Fig. 4e). In the *TBX3*/*TBX5* and the *HOXD* cluster, which has very low expression in myogenic cells, we looked at the expression of two and three cluster members, respectively, but did not see any significant differences in FSHD1 or FSHD2 myoblast and myotube cells in comparison to controls (data not shown).

The RRBS analysis also identified one additional cluster, the 5S rRNA cluster on chromosome 1, as being hypomethylated in the FSHD2 individuals. We quantified the expression of 5S rRNA, not only from the cluster as it is indistinguishable from other genome copies, in control, FSHD1, and FSHD2 myoblast and myotube cultures, and found that expression was almost significantly increased in FSHD2 myoblast cells (*Bonferroni adjusted *P* = 0.058) but was unchanged in myotubes (Fig. 4f, g). From mining publically available data sets (NCBI GEO: GSE65749 and GSE46462), there is not yet a clear indication for where SMCHD1 might bind within the 5S rRNA cluster.

We also examined the expression of the *HOXB*, the *HOXD*, the chromosome 1 tRNA, and the chromosome 1 5S rRNA clusters in the three 4qB SMCHD1 knockdown myoblast/myotube cell lines compared to control knockdown. We found no differences in cluster expression between the control and SMCHD1 knockdown cells (data not shown), except for at the chromosome 1 5S rRNA cluster in myoblast cells where expression was significantly increased with SMCHD1 knockdown (Fig. 4h).

These clusters highlight that hypomethylation seen at autosomal clusters in individuals carrying heterozygous *SMCHD1* mutations can affect the expression of a limited set of the genes in these regions but that hypomethylation does not necessarily result in an expression change.

## Discussion

SMCHD1 is an atypical member of the SMC protein family, with the ability to homodimerize and bind to DNA [6, 7, 38]. It has been established as an important player in epigenetic gene silencing, which is especially crucial in the case of FSHD2, where mutations in *SMCHD1* lead to loss of D4Z4 repeat silencing in somatic cells. Previous studies in mice have highlighted a role for Smchd1 in X inactivation and silencing at some clustered autosomal loci [11, 12]. In our study, we sought to further explore the silencing role of SMCHD1 in a human context by investigating the methylation changes occurring globally on autosomes in individuals with heterozygous *SMCHD1* mutations. By performing a 450k array on mononuclear monocytes and an RRBS analysis on primary muscle cell cultures, we assessed the resulting methylation changes. While FSHD is a muscle disease and we used peripheral blood cell DNA for the 450k arrays, previous studies have shown that DNA methylation levels at D4Z4 are comparable between hematogenous cells and myogenous cells [46]. Indeed, by and large, the same regions were identified as hypomethylated in our 450k methylation RRBS analysis. This is likely associated with the early establishment of DNA methylation during development, which is continually maintained in different tissue types. From our analysis, we found that the majority of all changes in autosomal methylation in heterozygous *SMCHD1* mutation carrying individuals were associated with gene clusters.

Most striking from our analysis was the extensive hypomethylation of the *PCDH* cluster on chromosome 5. A number of studies on Smchd1 function have been carried out in *Smchd1*-null (*Smchd1* ^MommeD1/MommeD1^) mice. These studies have highlighted the hypomethylation and transcriptional dysregulation of gene clusters caused by carrying homozygous Smchd1 mutations. Specifically, studies in *Smchd1*-null mouse embryos identified transcriptional upregulation of the clustered *Pcdh*α, *Pcdh*β, and *Pcdh*γ genes and showed that the promoters of some *Pcdh*α and *Pcdh*β genes were hypomethylated. Additionally, a recent study in mouse *Smchd1*-null neural stem cells found the *Pchd* cluster to be transcriptionally dysregulated, and SMCHD1 binding was identified at the *Pcdh*α cluster regulatory site HS5-1 [38]. Another study found that upon SMCHD1 knockdown in HEK293T and SK-SY5Y, some *PCDH*β genes were transcriptionally misregulated [40]. Furthermore, *Pcdh* cluster studies in mice have found that the locus is regulated by a number of DNAse1 HSs. HS5-1, positioned between the *Pchd*α and *Pcdh*β cluster, is known to regulate the *Pcdh*α cluster [47], while the CCR, consisting of six HS sites, is an enhancer region found downstream of the *Pchd*γ cluster and is important for the regulation of the *Pcdh*β cluster [41].

We identified binding of SMCHD1 to HS17-17′ and HS19-20 HS within the CCR, which showed a reduction in heterozygous *SMCHD1* mutation carriers associated with altered expression of some *PCDH*β isoforms, consistent with earlier studies in mice and HEK293 cells [40, 41]. Previous mouse studies implicated *Smchd1* in *Pcdh* cluster regulation through binding to the *Pcdh*α cluster HS5-1 regulatory site [38]; however, we could not confirm binding to this HS in human primary muscle cells. HS5-1 is a *cis*-regulatory site acting with opposite activity in neuronal cells versus non-neuronal cells, enhancer and repressor, respectively, in mice [47, 48]. This suggests that SMCHD1 has a tissue-specific binding pattern and/or differs in its binding pattern between species, as mouse ChIP studies were carried out in neuronal stem cells compared to the human muscle cells we investigated. In addition, the *PCDH*α cluster has little to no detectible transcription in myoblast cells (GEO GSE56787 from [42]) compared to the expressed *PCDH*β cluster; thus, other regulatory mechanisms may be at play at alpha regulatory sites in myoblast cells.

Previous studies, via knockout or shRNA modulation respectively, have found that reduction in SMCHD1 level results in transcriptional alteration of some clustered *Pcdh*/*PCDH* isoforms [38, 40]. Remarkably heterozygous mutations in *SMCHD1* lead to a loss of SMCHD1 binding at CCR regulatory sites in myoblast cells and resulted in an upregulation of some *PCDH*β cluster isoforms. This implicates SMCHD1 as a key repressor of the *PCDH*β cluster. We postulate that the long-range mode by which SMCHD1 is involved in *PCDH*β isoform expression regulation might be similar to that of the *PCDH*α DNA-looping model. In this model, *PCDH*α promoter activation occurs through DNA looping with the downstream HS5-1 enhancer where maximal activation occurs through contact with an active CTCF/cohesion transcriptional hub [49]. A recent mouse study showed that loss of Smchd1 leads to increased expression of certain *Pcdh*α isoforms and hypothesized that SMCHD1 participates in this HS5-1 transcriptional hub through either cooperative or competitive binding with CTCF to repress expression of certain *Pcdh*α isoforms [38]. Our results suggest that a similar regulatory mechanism is at play at the *PCDH*β enhancer sites, where loss of repressive SMCHD1 at these sites results in activation of certain *PDCH*β isoforms in muscle cells.

Protocadherins are predominantly, but not exclusively, expressed in the nervous system wherein different combinations of clustered *PCDH* genes are expressed in each neuron to create a repertoire of cell-surface diversity that is important for neuronal connectivity, self-recognition, and self-avoidance (reviewed in [50]). In addition, the clustered protocadherins have been suggested to play a role in signaling through binding with a number of signaling molecules [51, 52] and also potentially as proteolytically γ-secretase cleaved fragments that have the potential to enter the nucleus [53, 54]. Although we find hypomethylation of the *PCDH* cluster, this generally results in limited transcriptional changes, especially in myotube cells, which may be related to the decrease in SMCHD1 protein levels during muscle cell differentiation [55], or a shift in the transcriptional profile between the cell types. The biological significance of these transcriptional changes in muscle cells remains to be established.

We also identified SMCHD1 as a regulator of the tRNA cluster on chromosome 1, with *SMCHD1* mutations resulting in hypomethylation and gene expression upregulation within the cluster. Additionally, we confirmed a putative SMCHD1 binding site within the tRNA cluster but did not see a reduction in binding to this site in FSHD2 individuals. The tRNA cluster on chromosome 1 is known to have very high CG content (64.2%) and display extensive copy number variation, and in addition, it is marked with histone H3 trimethylated at lysine 4 (H3K4me3) and histone H3 trimethylated at lysine 9 (H3K9me3), giving it a sequence structure very similar to that of the D4Z4 locus [45]. The absence of a significant loss in binding in FSHD2 individuals may be due to the extensive size polymorphisms at this locus [44, 45], for which we do not have information on for any individual. How or whether tRNA dysregulation could contribute to FSHD2 pathophysiology is unknown. The function of tRNAs is varied, with their main purpose being their role in translation. However, there are an increasing number of studies highlighting alternative functions for both tRNAs and tRNA fragments in signaling and gene regulation (reviewed in [56, 57]).

## Conclusions

Taken together, our results highlight a role for SMCHD1 in the regulation of a limited number of human autosomal gene clusters, aside from its repressive role at the D4Z4 repeat. Heterozygous mutations in *SMCHD1* lead to hypomethylation at these autosomal gene clusters, insinuating a role for methylation maintenance at these loci and proving that a heterozygous mutation is on its own capable of conferring methylation changes. Our results largely identify clusters from homozygous *Smchd1* knockout mouse studies but identify two new clusters, the tRNA and 5S rRNA clusters on chromosome 1, as being SMCHD1 regulated. Furthermore, we find that SMCHD1 acts as a repressor at the clustered *PCDH*β genes in muscle cells and at the tRNA cluster on chromosome 1, where we confirmed SMCHD1 binding at both loci and show that a reduction of SMCHD1 binding or reduced methylation results in expression changes for some isoforms of the clusters. However, the gene expression changes are generally limited, especially in myotubes where there is concomitant reduction in SMCHD1 protein levels [55]. Perhaps consistent with the small changes, SMCHD1 mutation carriers show no striking phenotype aside from FSHD, and in addition, there is no overt phenotype in SMCHD1 mutation carriers with two 4qB chromosomes. Thus, at this point, it appears these additional alterations in methylation and expression likely do not result in an obvious phenotype on their own; however, there still exists the possibility that they may contribute to the FSHD phenotype in an unknown way. These findings are encouraging for the further exploration into FSHD therapies aimed at modulating SMCHD1 levels, as these studies point to a relatively minimal number of autosomal loci outside the D4Z4 repeat array that are strongly influenced by SMCHD1 levels.

## Additional files

Additional file 1: Table S1. Summary of material used in the studies. (PDF 43 kb)

Additional file 2: Table S2. Gene-specific primers used for ChIP and qPCR analysis. (PDF 25 kb)

Additional file 3: Table S3. Differentially methylated regions identified by 450k. (PDF 48 kb)

Additional file 4: Table S4. Differentially methylated regions identified by RRBS. (PDF 170 kb)

Additional file 5: Figure S1. Methylation of the *TCEB3C* cluster and *ZAV* macrosatellite in FSHD2 and control individuals. Relative methylation levels found in FSHD2 and control individuals by RRBS, displayed as a methylation heatmap with color scale from yellow, no/low number of methylated CpGs at the locus to dark blue, high number of methylated CpGs at (A) the *TCEB3C* cluster and (B) the *ZAV* macrosatellite in primary myoblast cells. Bisulfite sequencing of (C) the *TCEB3C* cluster displayed as a heatmap depicting the average methylation fraction at a specific CpG from ESME analysis, with color scale from yellow to dark blue indicating a low to high percent of methylation, and (D) the *ZAV* macrosatellite depicted as single molecule clones for individuals, where black circles represent a methylated cytosine and open circles an unmethylated cytocine; gender of the individual is indicated by a red line for females and blue line for males in peripheral blood mononuclear cells. (PDF 109 kb)

Additional file 6: Figure S2. Transcriptional expression of the *PCDH*γ cluster in control, FSHD1, and FSHD2 individuals. RNA expression analysis of *PCDH*γ cluster isoform members in primary (A) myoblast cells and (B) myotube cells. Results represent log10 relative expression by qRT-PCR analysis of the indicated gene after normalization to the internal control gene *GUS1*. For each gene, the value of expression in control individuals was then arbitrarily set to 1. *Bonferroni adjusted *P* < 0.05, *t* test; *n* = 4 for controls, *n* = 10 for FSHD1, *n* = 7 for FSHD2; error bars = SEM. (PDF 1031 kb)

Additional file 7: Figure S3. DUX4 binding at HS16. UCSC genome track of aligned DUX4 ChIP-sequencing reads from a previously published study (GEO GSE33838 [43]) showing a peak of DUX4 binding at HS16 within the cluster control region of the PCDH cluster. Sequence in the peak region is listed highlighting the DUX4 consensus binding sequence in red. (PDF 151 kb)

Additional file 8: Figure S4. Transcriptional expression of DUX4 target genes and the *PCDH*γ cluster in SMCHD1 KD 4qB cell lines. RNA expression analysis of (A) the DUX4 target genes *ZSCAN4* and *LEUTX* in primary myotubes and the *PCDH*γ cluster isoform members in primary (B) myoblast and (C) myotube cells after SMCHD1 shRNA KD or control shRNA KD targeting luciferase and GFP. Results represent qRT-PCR analysis of the indicated gene after normalization to the internal control gene *GUS1*. For each gene, the value of expression in control shRNA samples was then arbitrarily set to 1. *Bonferroni adjusted *P* < 0.05, *t* test; *n* = 6 (two shRNA constructs for each target on three independent cell lines); error bars = SEM. (PDF 1017 kb)

Additional file 9: Figure S5. Percent input ChIP-qPCR for representative individuals. ChIP-qPCR for all ChIP examined loci displayed as a percent of the input for one control, FSHD1, and FSHD2 individual. Please note that it is difficult to compare single copy loci, HS17-17′and HS19-20, to multi-copy loci tRNA and D4Z4. (PDF 831 kb)

## Abbreviations

CCR: Cluster control region
ChIP-qPCR: Chromatin immunoprecipitation followed by quantitative PCR
Cs: Cytosines
DMB: Differential methylation block
DMR: Differentially methylated region
DNMT3B: DNA methyltransferase 3B
DUX4: Double homeobox 4
FSHD: Facioscapulohumeral muscular dystrophy
PAS: Polyadenylation signal
*PCDH*/Pchd: Protocadherin
RRBS: Reduced representation bisulfite sequencing
SMC: Structural maintenance of chromosomes
SMCHD1: Structural maintenance of chromosomes flexible hinge domain containing 1
