# Supplementary material for: SMCHD1 regulates a limited set of gene clusters on autosomal chromosomes
Source: Skelet Muscle. 2017 Jun 6;7:12. doi: 10.1186/s13395-017-0129-7 (PMC5461771; doi:10.1186/s13395-017-0129-7)
Supplement: Supplementary file 1 — Summary of material used in the studies. (PDF 43 kb) [file 13395_2017_129_MOESM1_ESM.pdf]

ID	Rf.Nr	M/F	SMCHD1 mutationcDNA (NM_015295.2)	status	450K array	RRBS	qPCR	ChIP
w412	Rf7.204	F	none	Control	x			
w1848	Rf28.102	F	none	Control	x			
r450	Rf204.306	F	none	Control	x			
r74	Rf208.318	F	none	Control	x			
r285	Rf209.210	F	none	Control	x			
r352	Rf210.104	F	none	Control	x			
r63	Rf268.1	F	none	Control	x			
w1825	Rf300.5	F	none	Control	x			
r264	Rf385.302	F	none	Control	x			
r322	Rf400.2	F	none	Control	x			
r650	Rf649.4	F	none	Control	x			
r860	Rf739.3	F	none	Control	x			
b59	Rf696.2	F	none	Control	x			
r795	Rf742.3	F	none	Control	x			
r977	Rf844.4	F	none	Control	x			
b46	Rf909.2	F	none	Control	x			
r997	Rf910.3	F	none	Control	x			
b61	Rf952.2	F	none	Control	x			
b93	Rf969.1	F	none	Control	x			
b323	Rf1021.5	F	none	Control	x			
b288	Rf1110.2	F	none	Control	x			
b480	Rf1126.202	F	none	Control	x			
b567	Rf1232.4	F	none	Control	x			
b609	Rf629.2	F	c.5602C>T	Mutation carrier	x			
b227	Rf393.302	F	c.1608del	Mutation carrier	x			
r272	Rf385.102	F	c.5596C>G	Mutation carrier	x			
r77	Rf399.3	F	c.2068C>T	Mutation carrier	x			
r837	Rf683.1	F	c.4661T>C	Mutation carrier	x	x	x	x
r861	Rf743.2	F	c.182_183dup	Mutation carrier	x			
r864	Rf744.4	F	unknown	Mutation carrier	x			
r893	Rf562.1	F	c.2191C>T	Mutation carrier	x			
r944	Rf874.3	F	c.3274_3276+1del	Mutation carrier	x			
r950	Rf878.2	F	c.3276+4_3276+7del	Mutation carrier	x			
r973	Rf649.3	F	c.3801+1G>A	Mutation carrier	x			
b13	Rf898.2	F	1,2 Mb deletion SMCHD1	Mutation carrier	x			
b17	Rf922.2	F	c.412C>T	Mutation carrier	x			
b37	Rf936.1	F	c.3276_3276+4del	Mutation carrier	x			
b109	Rf975.201	F	c.24del	Mutation carrier	x			
b206	Rf1033.1	F	c.1302_1306delTGATA	Mutation carrier	x		x	x
b260	Rf1021.2	F	c.1580C>T	Mutation carrier	x			
b286	Rf1110.3	F	c.3048+2T>C	Mutation carrier	x			
b376	Rf392.102	F	c.4566G>A	Mutation carrier	x			
b398	Rf1014.4	F	c.4566G>A	Mutation carrier	x			
b454	Rf1101.2	F	c.582dupT	Mutation carrier	x			
b483	Rf1126.204	F	c.4404G>A	Mutation carrier	x			
b493	Rf1196.1	F	c.1273G>A	Mutation carrier	x			
b565	Rf909.206	F	c.2665dupA	Mutation carrier	x			
r730	Rf745	F	none	Control		x	x	x
b118	Rf854	M	none	Control		x		
r870	Rf844	M	c.3274_3276+1del	Mutation carrier		x	x	x
b194	Rf1024	M	none	Control			x	x
v96	Rf899	M	none	Control			x	x
b887	Rf943	F	none	Control			x	x
b735	Rf1033	F	c.1302_1306delTGATA	Mutation carrier			x	x
b206	Rf1033	F	c.1302_1306delTGATA	Mutation carrier			x	x
r648	Rf696	M	c.1647+3A>G	Mutation carrier			x	x
r887P	Rf854	M	c.3444T>A	Mutation carrier			x	
r562	Rf645	F	none	Contracted D4Z4			x	x
r707	Rf731	F	none	Contracted D4Z4			x	x
r473	Rf584	F	none	Contracted D4Z4			x	
r603	Rf675	F	none	Contracted D4Z4			x	
r738	Rf752	F	none	Contracted D4Z4			x	
r719	Rf737	M	none	Contracted D4Z4			x	
b22	Rf925	F	none	Contracted D4Z4			x	
r966	Rf887	F	none	Contracted D4Z4			x	
r937	Rf870	M	none	Contracted D4Z4			x	
r218	Rf353	F	none	Contracted D4Z4			x	

Cells which contain two 4QB alleles used for SMCHD1 KD

ID	Rf.Nr	M/F	SMCHD1 mutationcDNA (NM_015295.2)	4q allele 1	4q allele 2
r879	Rf852	M	none	B	B
b194	Rf1024	M	none	B	B
b94	Rf964	F	none	B	B
