# Supplementary material for: SMCHD1 regulates a limited set of gene clusters on autosomal chromosomes
Source: Skelet Muscle. 2017 Jun 6;7:12. doi: 10.1186/s13395-017-0129-7 (PMC5461771; doi:10.1186/s13395-017-0129-7)
Supplement: Supplementary file 2 — Gene-specific primers used for ChIP and qPCR analysis. (PDF 25 kb) [file 13395_2017_129_MOESM2_ESM.pdf]

ChIP-qPCR primers	
HS5-1 F	CCTGGTTTCTGCCGTTCTAA
HS5-1 R	GCACTGGGACGGACTACATT
HS17-17' F	CAAGGTCCATTACGCCACCT
HS17-17' R	AGGGTTTGTCCCCAGGTACT
HS19-20 F	TGCTTTACTGCTTTCAGCCA
HS19-20 R	ACCAAACGCAACAATGACACAT
tRNA F	GCCATTACTACCAAAGCCGC
tRNA R	ATCTGCGCCTAATGGTTGGG

Gene Expression Primers	
GUS1 F	CTCATTTGGAATTTTGCCGATT
GUS1 R	CCGAGTGAAGATCCCCTTTTTA
PCDHB2 F	AACGGAGAGTGGCTCCTTTG
PCDHB2 R	CTAATCCGTAGCTCCGCCTG
PCDHB3 F	GGTTAGCGAGGCAAATCCCA
PCDHB3 R	CTTGCCTTGAACCAGACCAAA
PCDHB5 F	GGTGTGTTTGACCGGAGACT
PCDHB5 R	TTCCGGAAGGCAGCAGTTTT
PCDHB6 F	ACGCCCTGTTTCAAGTCGAT
PCDHB6 R	CATGGTGAGTTCAGGGGCAT
PCDHB7 F	ACGACATCTGGTGGACTTGAG
PCDHB7 R	CTGTGCTCTGGGGTAGCAGG
PCDHB8 F	ATCAGTACGAGGTGTGCCTG
PCDHB8 R	AGGCTGAAACCAAAGCCATTC
PCDHB9 F	ACCCTGTTCCAGAGCTACCA
PCDHB9 R	TCCTCTATTTCTTTCCCACCCC
PCDHB10 F	TTTTCCAGGGCATCTGGTGG
PCDHB10 R	CCCTGTGCCTGAATATCCGAA
PCDHB11 F	AAATACGCCAGAGACCGTGG
PCDHB11 R	CACAGTTGTGTTGTGCTCGG
PCDHB12 F	GACATCTGGTGGACGTGAGTG
PCDHB12 R	ACCTGTGCTCTGGGGTAGGAA
PCDHB13 F	AGTTCAAGTTCCTGAAGCCGA
PCDHB13 R	AACCCAAAGTTATTGGGGAAGG
PCDHB14 F	GTGTCTGACAGGAGGTTCCG
PCDHB14 R	CGAAAGTTCTCGATTTCCCCC
PCDHB15 F	CTGACGGGAGGCTCTGAAAG
PCDHB15 R	ACAGACGGTCGGAAAGCTAC
PCDHB16 F	CCAGGAAGACCTTCCCTTTC
PCDHB16 R	GGTGTAGGAGGTTTGGGTGA
PCDHB18 F	CCAAAGGTCCAGGCTGATAGG
PCDHB18 R	CCGGCTCCATAGCTCCTTAG
PCDHGA1 F	CCCAACTATGCGGACACACT
PCDHGA1 R	AGCTGATACATTCATCACAAAAGTT
PCDHGA2 F	ATTCAGTCGAACAGCCCACC
PCDHGA2 R	TTTTGCAGAGCCGCCATTTC
PCDHGA3 F	TTCCGAAATGGCAGAGGACT
PCDHGA3 R	CCCAGGTCGTTAGCGATGTT
PCDHGA4 F	CCAACCCAGCTATGCAGACA
PCDHGA4 R	TGAGAGACATTTACACAACTCTTTG
PCDHGB1 F	CAACCTGACACCGGAAATGG
PCDHGB1 R	GCAGAAACTCACGTGCGAAG
PCDHGB2 F	TTGTCACCCTGCACATCTCC
PCDHGB2 R	GCCACGTGAACCATGTAGGA
PCDHGB3 F	CTCCCCACCTACAGCGAAAG
PCDHGB3 R	GGAAAGGTTTGAAGAAACTCACCT

PCHDGB5 F	TGAGCTCCGGATCCAGGTAA
PCHDGB5 R	TGCCCGGTTCTGTAGAAGGA
PCDHGB7 F	CTCCCAGCGTTGAAGCAGAT
PCDHGB7 R	GGTGTCATCGCCATTTTGGG
PCDHGC3 F	CCGGTGTTCTATAGGCAGGTG
PCDHGC3 R	AGAAACGCCAGTCCGTGTT
PCDHGC5 F	ACAGGACGTGCTTTTCACCG
PCDHGC5 R	GTGTCATCGCCATTTTGGGAG
HOXB3 F	GCCATTCTGTGTAGACAAGAGC
HOXB3 R	ATCTCCCCTCCTTTGCGCC
HOXB4 F	AGCACGGTAAACCCCAATTACG
HOXB4 R	GTGTCAGGTAGCGGTTGTAGT
HOXB5 F	ACATCAGCCATGATATGACCGGG
HOXB5 R	TCAGGTAGCGGTTGAAGTGG
HOXB6 F	ATTCGTGCAACAGTTCCTCCT
HOXB6 R	CGCGTCAGGTAGCGATTGTA
HOXB7 F	AGCTCAGGAACTGACCGCAAA
HOXB7 R	CCTGTCTTGGCCGGTGGTC
tRNA_GlyTCC F	GTCAACTGCTTGGAAGG
tRNA_GlyTCC R	CGTTGGTGGTATAGTGGTGA
tRNA_ Glu CTC/TCC F	CTGACCGGGAATCGA
tRNA_ Glu CTC/TCC R	CTCCCTGGTGGTCTAGT
tRNA_AspGTC F	TCCCCGTCGGGGAATCGA
tRNA_AspGTC R	TCCTCGTTAGTATAGTGGT
tRNA_ LeuCAG F	CAGGATGGCCGAGCGGT
tRNA_ LeuCAG R	TCAGGAGTGGGATTCGAAC
tRNA_GlyGCC F	CATGGGTGGTTCAGTGGTAGA
tRNA_GlyGCC R	GGGAATCGAACCCGGGCCTC
tRNA_ValCAC F	GTTTCCGTAGTGTAGTGGTATTC
tRNA_ValCAC R	TTTCCGCCCGGTTTCGAAC
5S_rRNA F*	GGCCATACCACCCTGAACGC
5S_rRNA R*	CAGCACCCGGTATTCCCAGG

*C.Liu et al. 2015, Nucleic Acids Res

Methylation Specific primers

TCEB3C_F	GATGGGGGTYGGAATTAGTTGGGGTTATTTTGGG
TCEB3C_R	AAACACCTCCRAAACAACAAAAACCTCACCC
ZAV_F	TTTTGTTTAYGATTAGTTAGGTTTGAGTTG
ZAV_R	TACCTAAAATCRTATACCCCTTCCTTAATTCAC
