# Supplementary material for: SMCHD1 regulates a limited set of gene clusters on autosomal chromosomes
Source: Skelet Muscle. 2017 Jun 6;7:12. doi: 10.1186/s13395-017-0129-7 (PMC5461771; doi:10.1186/s13395-017-0129-7)
Supplement: Supplementary file 3 — Differentially methylated regions identified by 450k. (PDF 48 kb) [file 13395_2017_129_MOESM3_ESM.pdf]

chrom.	start	end	Nearest gene	Effect size	#CpGs
chr2	176982108	176982342	HOXD10 (+733)	-0.061011884	3
chr3	147077186	147077671	PLSCR1 (-814,801), ZIC4 (+44,642)	0.032308901	7
chr3	147111280	147112143	PLSCR1 (-849,084), ZIC4 (+10,359)	0.027142669	6
chr3	147131069	147131617	ZIC1 (+4,162)	0.026977624	5
chr3	147142182	147142355	ZIC1 (+15,088)	0.054174877	3
chr5	1962311	1962554	IRX4 (-79,553), IRX2 (+789,336)	-0.129595712	3
chr5	42944267	42944494	SEPP1 (-132,357), C5orf39 (+96,066)	0.063034933	3
chr5	140174449	140174887	PCDHAC2 (-171,079), ZMAT2 (+94,636)	-0.086167593	5
chr5	140180691	140181074	PCDHAC2 (-164,864), ZMAT2 (+100,851)	-0.064843346	5
chr5	140213825	140214328	PCDHAC2 (-131,670), ZMAT2 (+134,045)	-0.076618858	5
chr5	140248564	140249080	PCDHAC2 (-96,925), ZMAT2 (+168,790)	-0.110398562	5
chr5	140305947	140306458	PCDHAC2 (-39,544), ZMAT2 (+226,171)	-0.032544589	10
chr5	140474005	140475652	PCDHB2 (+592)	-0.090184933	13
chr5	140502903	140503914	PCDHB5 (-11,391), PCDHB4 (+1,828)	-0.079232952	6
chr5	140517239	140517411	PCDHB6 (-12,514), PCDHB5 (+2,525)	-0.107149392	3
chr5	140529499	140530028	PCDHB6 (-75)	-0.072071447	7
chr5	140530410	140532058	PCDHB17 (-4,346)	-0.104055883	8
chr5	140535215	140536496	PCDHB17 (+276)	-0.103731987	10
chr5	140536920	140537705	PCDHB7 (-14,930), PCDHB17 (+1,733)	-0.095577312	3
chr5	140553091	140554819	PCDHB8 (-3,416)	-0.093035569	7
chr5	140559576	140559871	PCDHB16 (-1,256)	-0.090130314	3
chr5	140562034	140562819	PCDHB9 (-4,466)	-0.104749247	6
chr5	140568217	140569144	PCDHB10 (-3,271)	-0.076468231	6
chr5	140573574	140574095	PCDHB11 (-5,513), PCDHB10 (+1,883)	-0.052873883	3
chr5	140595009	140595879	PCDHB14 (-7,634), PCDHB13 (+1,935)	-0.092306012	5
chr5	140614727	140615656	PCDHB15 (-9,955), PCDHB18 (+1,254)	-0.090836751	5
chr5	140625848	140627399	PCDHB15 (+1,477), SLC25A2 (+56,988)	-0.078093722	5
chr5	140700583	140700662	TAF7 (-272)	-0.032718164	4
chr5	140718339	140719142	PCDHGC5 (-150,067), TAF7 (-18,390)	-0.066598696	6
chr5	140719962	140720426	PCDHGC5 (-148,614), TAF7 (-19,843)	-0.076945021	4
chr5	140723509	140725147	PCDHGC5 (-144,480), TAF7 (-23,977)	-0.076660868	12
chr5	140729653	140730092	PCDHGC5 (-138,935), TAF7 (-29,522)	-0.084473646	5
chr5	140731308	140731930	PCDHGC5 (-137,189), TAF7 (-31,268)	-0.066408947	3
chr5	140734721	140735397	PCDHGC5 (-133,749), TAF7 (-34,708)	-0.053784962	6
chr5	140736762	140737111	PCDHGC5 (-131,871), TAF7 (-36,586)	-0.071199968	5
chr5	140739522	140740387	PCDHGC5 (-128,853), TAF7 (-39,604)	-0.096161819	5
chr5	140743927	140744367	PCDHGC5 (-124,661), TAF7 (-43,796)	-0.061752996	4
chr5	140745340	140746162	PCDHGC5 (-123,057), TAF7 (-45,400)	-0.070024992	5
chr5	140749735	140750160	PCDHGC5 (-118,860), TAF7 (-49,597)	-0.069151666	6
chr5	140751454	140751608	PCDHGC5 (-117,277), TAF7 (-51,180)	-0.085659405	3
chr5	140753415	140753924	PCDHGC5 (-115,138), TAF7 (-53,319)	-0.074974007	10
chr5	140761259	140764302	PCDHGC5 (-106,027), TAF7 (-62,430)	-0.108179338	10
chr5	140767200	140769440	PCDHGC5 (-100,488), TAF7 (-67,969)	-0.083170482	14
chr5	140772182	140772859	PCDHGC5 (-96,287), TAF7 (-72,170)	-0.111259103	7
chr5	140773864	140774440	PCDHGC5 (-94,656), TAF7 (-73,801)	-0.131794116	4
chr5	140777344	140779461	PCDHGC5 (-90,405), TAF7 (-78,052)	-0.100285737	13
chr5	140782297	140783173	PCDHGC5 (-86,073), TAF7 (-82,384)	-0.086243068	6
chr5	140787430	140788504	TAF7 (-87,616), PCDHGC5 (-80,841)	-0.048633702	6
chr5	140789247	140789745	TAF7 (-89,145), PCDHGC5 (-79,312)	-0.094304829	4
chr5	140792558	140793307	TAF7 (-92,582), PCDHGC5 (-75,875)	-0.059267648	8
chr5	140794664	140794993	TAF7 (-94,478), PCDHGC5 (-73,979)	-0.134057819	3
chr5	140797509	140799572	TAF7 (-98,190), PCDHGC5 (-70,267)	-0.111293932	10
chr5	140801354	140802831	TAF7 (-101,742), PCDHGC5 (-66,715)	-0.102576925	7
chr5	140810109	140811253	TAF7 (-110,330), PCDHGC5 (-58,127)	-0.096306705	13
chr5	140892308	140893634	PCDHGC5 (+24,163), DIAPH1 (+105,651)	0.096740316	5
chr6	379489	379581	IRF4 (-12,204), DUSP22 (+87,434)	-0.09868766	3
chr7	57927758	57927793	ZNF716 (+417,893)	-0.059886956	3
chr7	63151988	63153149	LOC643955 (-388,135), ZNF727 (-353,252)	-0.03772025	3

chr7	63361127	63361617	LOC643955 (-596,938), ZNF727 (-144,449)	-0.091046653	5
chr7	63385990	63387147	LOC643955 (-622,135), ZNF727 (-119,252)	-0.082389573	7
chr8	22560972	22561719	EGR3 (-10,531), PEBP4 (+224,075)	0.055866628	6
chr8	144360667	144362100	ZNF696 (-12,175), GLI4 (+11,777)	-0.123147931	4
chr8	144371537	144371779	ZNF696 (-1,901)	-0.157532087	3
chr12	114841202	114841870	RBM19 (-437,360), TBX5 (+2,353)	0.033705495	6
chr12	115122028	115122157	TBX3 (-124)	-0.010921959	5
chr13	36052768	36053408	MAB21L1 (+462,294), NBEA (+536,664)	0.04949918	4
chr13	112838022	112838611	SPACA7 (-192,334), SOX1 (+116,404)	-0.105336476	6
chr14	61748243	61748354	TMEM30B (+231)	0.055035436	3
chr15	23157727	23157832	GOLGA8IP (-97,462), NIPA1 (-71,344)	0.064704265	3
chr15	96873850	96874050	NR2F2 (-161)	-0.01625825	8
chr15	96904723	96905576	NR2F2 (+31,039)	0.042171742	5
chr16	32822612	32823584	TP53TG3C (-135,650), SLC6A10P (+68,824)	-0.079642512	4
chr16	32857347	32858241	TP53TG3C (-170,346), SLC6A10P (+34,128)	-0.09859056	5
chr16	33039897	33040275	TP53TG3B (-165,499), SLC6A10P (-148,164)	-0.07908879	3
chr16	83171068	83171314	HSBP1 (-670,402), CDH13 (+510,792)	-0.156549444	4
chr17	25289961	25290310	WSB1 (-330,970)	-0.17853885	3
chr17	46682308	46682394	HOXB6 (-17)	-0.11963952	4
chr19	51227943	51228937	GPR32 (-45,418), CLEC11A (+1,835)	-0.019204144	5
chr19	54515169	54515341	CACNG6 (+19,713), VSTM1 (+51,952)	0.08593187	4
chr22	16867554	16868045	OR11H1 (-417,996), CCT8L2 (+205,900)	-0.061636861	4
