# Supplementary material for: SMCHD1 regulates a limited set of gene clusters on autosomal chromosomes
Source: Skelet Muscle. 2017 Jun 6;7:12. doi: 10.1186/s13395-017-0129-7 (PMC5461771; doi:10.1186/s13395-017-0129-7)
Supplement: Supplementary file 4 — Differentially methylated regions identified by RRBS. (PDF 170 kb) [file 13395_2017_129_MOESM4_ESM.pdf]

Chromosome	Start	End	Width	Number of CpGs	Cluster Name
Chr.10	1.35E+08	1.35E+08	21037	649	**D4Z4 chr10**
Chr.4	1.91E+08	1.91E+08	27211	666	**D4Z4 chr4**
Chr.5	1.41E+08	1.41E+08	42104	342	**PCDH cluster**
Chr.1	2.29E+08	2.29E+08	39472	1369	**rRNA cluster**
Chr.1	1.61E+08	1.61E+08	31348	825	**tRNA cluster**
