# Supplementary figures and images for: SMCHD1 regulates a limited set of gene clusters on autosomal chromosomes
Source: Skelet Muscle. 2017 Jun 6;7:12. doi: 10.1186/s13395-017-0129-7 (PMC5461771; doi:10.1186/s13395-017-0129-7)

A

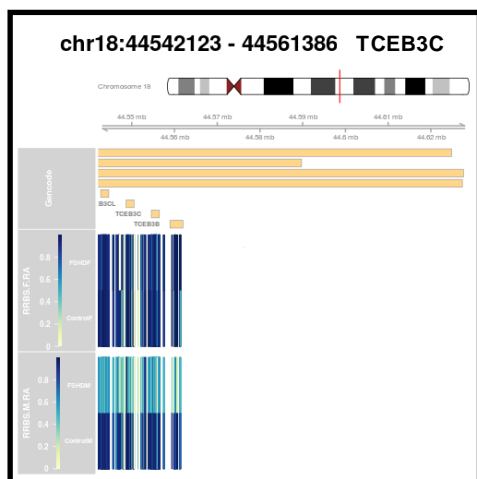

B

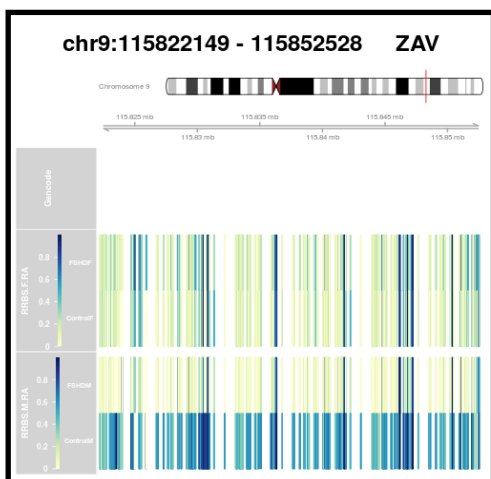

C

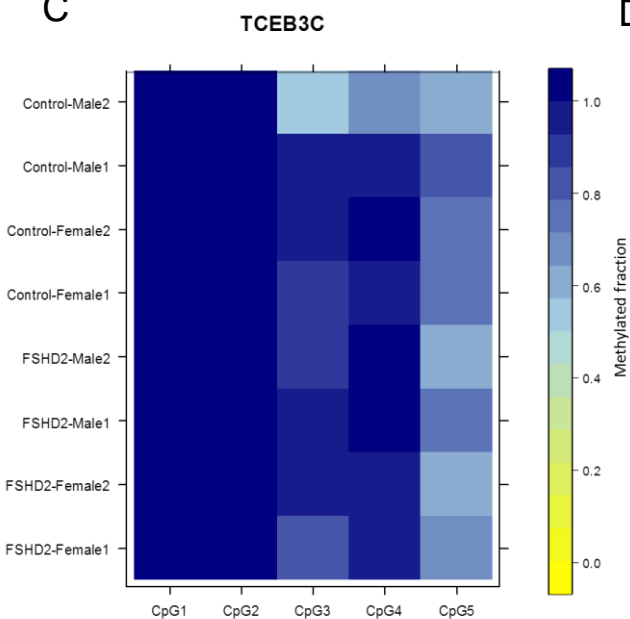

D

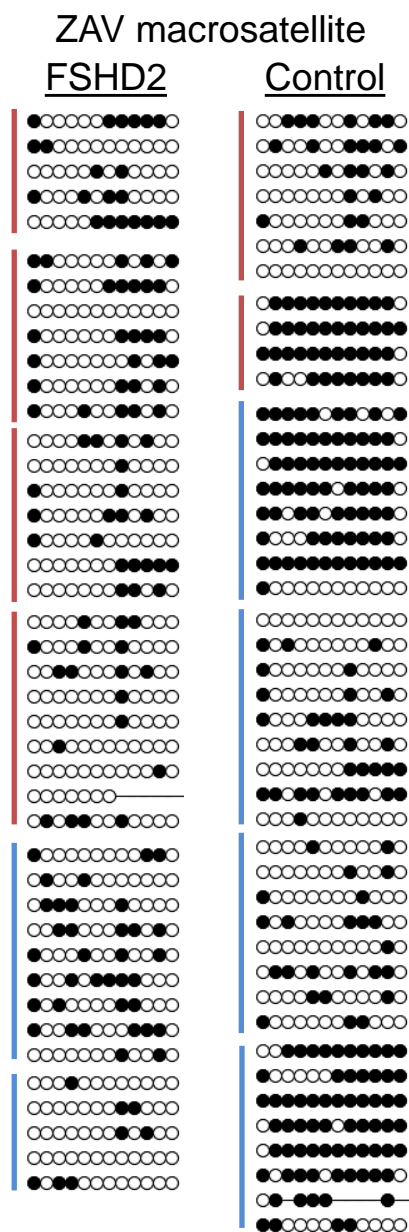

Supplement: Supplementary file 5 — Methylation of the TCEB3C cluster and ZAV macrosatellite in FSHD2 and control individuals. Relative methylation levels found in FSHD2 and control individuals by RRBS, displayed as a methylation heatmap with color scale from yellow, no/low number of methylated CpGs at the locus to dark blue, high number of methylated CpGs at (A) the TCEB3C cluster and (B) the ZAV macrosatellite in primary myoblast cells. Bisulfite sequencing of (C) the TCEB3C cluster displayed as a heatmap depicting the average methylation fraction at a specific CpG from ESME analysis, with color scale from yellow to dark blue indicating a low to high percent of methylation, and (D) the ZAV macrosatellite depicted as single molecule clones for individuals, where black circles represent a methylated cytosine and open circles an unmethylated cytocine; gender of the individual is indicated by a red line for females and blue line for males in peripheral blood mononuclear cells. (PDF 109 kb) [file 13395_2017_129_MOESM5_ESM.pdf]

A

# Myoblast

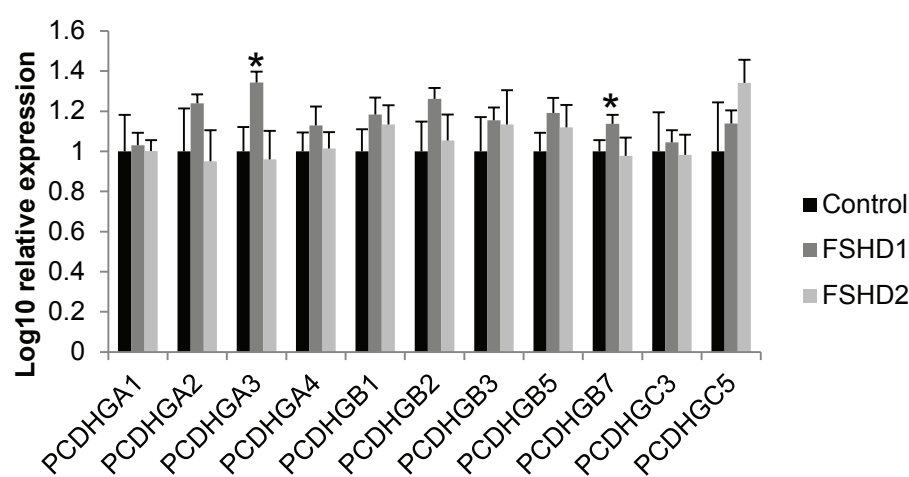

B

# Myotube

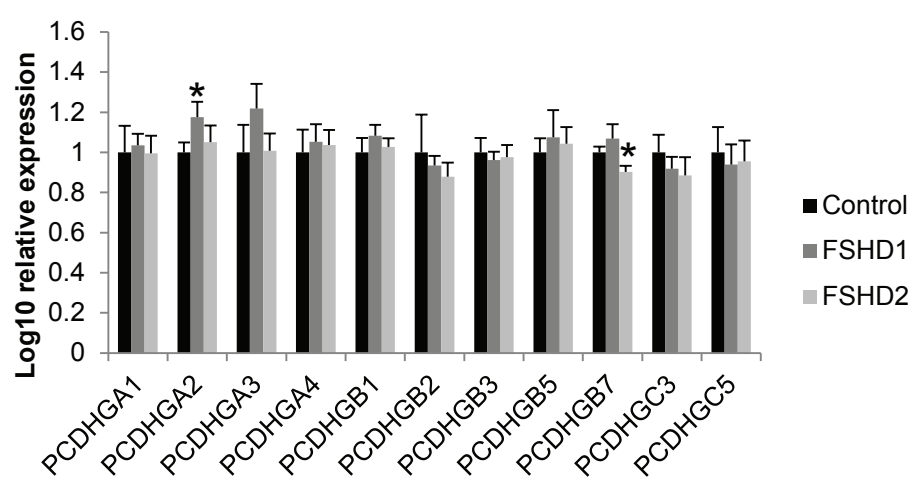

Supplement: Supplementary file 6 — Transcriptional expression of the PCDHγ cluster in control, FSHD1, and FSHD2 individuals. RNA expression analysis of PCDHγ cluster isoform members in primary (A) myoblast cells and (B) myotube cells. Results represent log10 relative expression by qRT-PCR analysis of the indicated gene after normalization to the internal control gene GUS1. For each gene, the value of expression in control individuals was then arbitrarily set to 1. *Bonferroni adjusted P < 0.05, t test; n = 4 for controls, n = 10 for FSHD1, n = 7 for FSHD2; error bars = SEM. (PDF 1031 kb) [file 13395_2017_129_MOESM6_ESM.pdf]

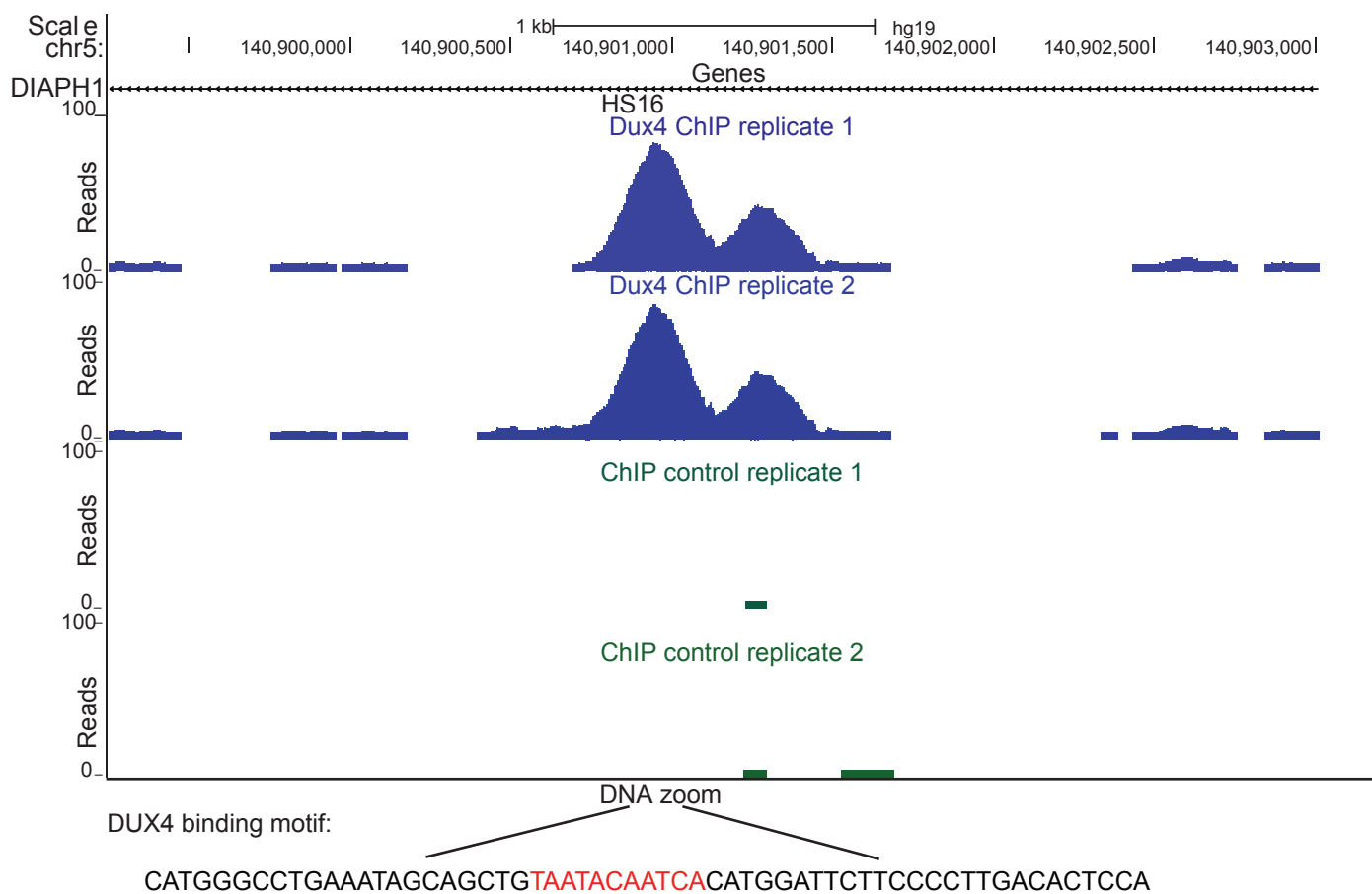

Supplement: Supplementary file 7 — DUX4 binding at HS16. UCSC genome track of aligned DUX4 ChIP-sequencing reads from a previously published study (GEO GSE33838 [43]) showing a peak of DUX4 binding at HS16 within the cluster control region of the PCDH cluster. Sequence in the peak region is listed highlighting the DUX4 consensus binding sequence in red. (PDF 151 kb) [file 13395_2017_129_MOESM7_ESM.pdf]

A

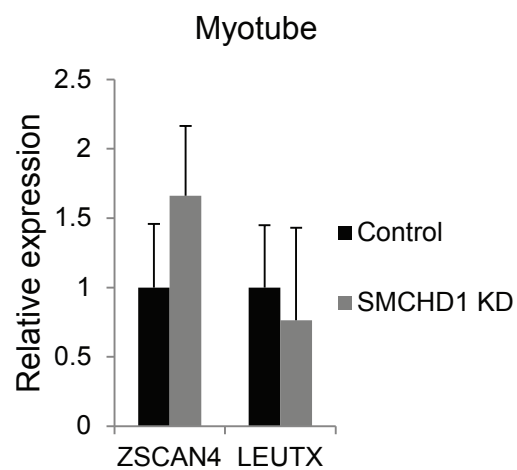

B

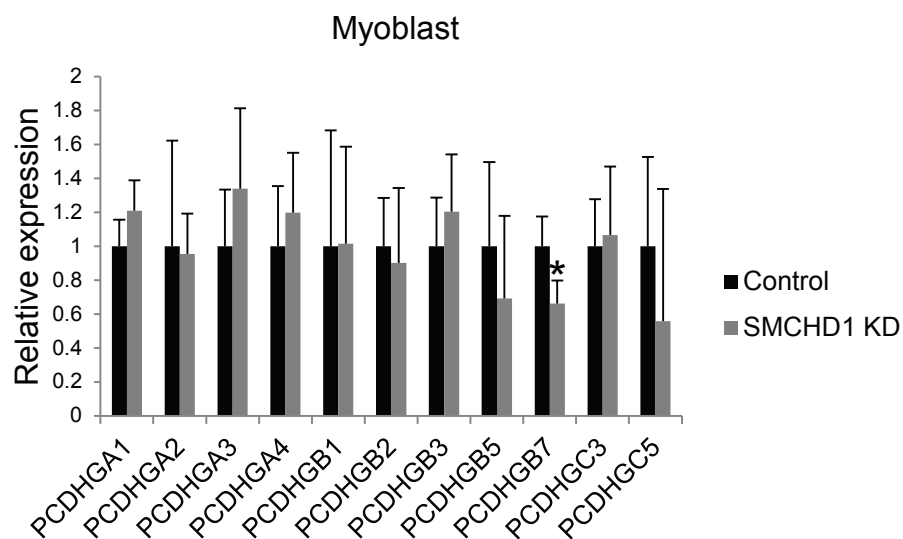

C

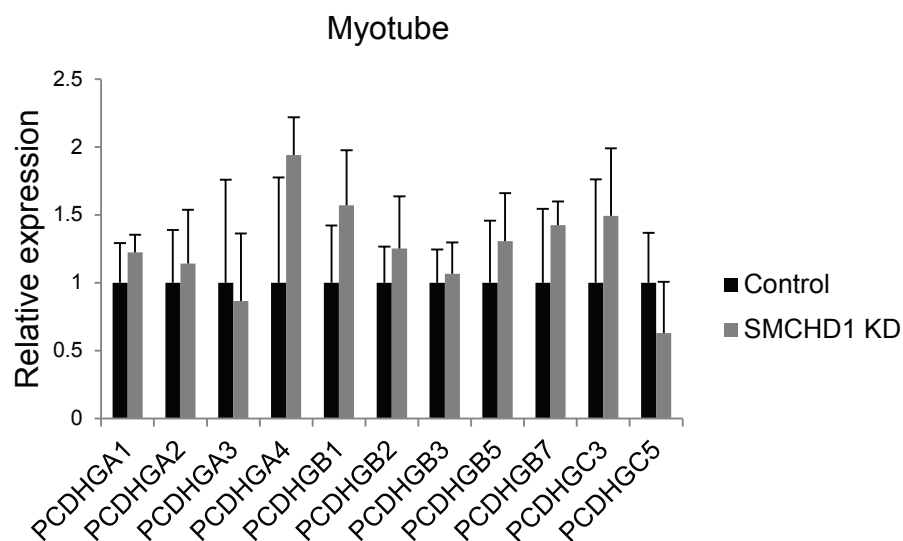

Supplement: Supplementary file 8 — Transcriptional expression of DUX4 target genes and the PCDHγ cluster in SMCHD1 KD 4qB cell lines. RNA expression analysis of (A) the DUX4 target genes ZSCAN4 and LEUTX in primary myotubes and the PCDHγ cluster isoform members in primary (B) myoblast and (C) myotube cells after SMCHD1 shRNA KD or control shRNA KD targeting luciferase and GFP. Results represent qRT-PCR analysis of the indicated gene after normalization to the internal control gene GUS1. For each gene, the value of expression in control shRNA samples was then arbitrarily set to 1. *Bonferroni adjusted P < 0.05, t test; n = 6 (two shRNA constructs for each target on three independent cell lines); error bars = SEM. (PDF 1017 kb) [file 13395_2017_129_MOESM8_ESM.pdf]

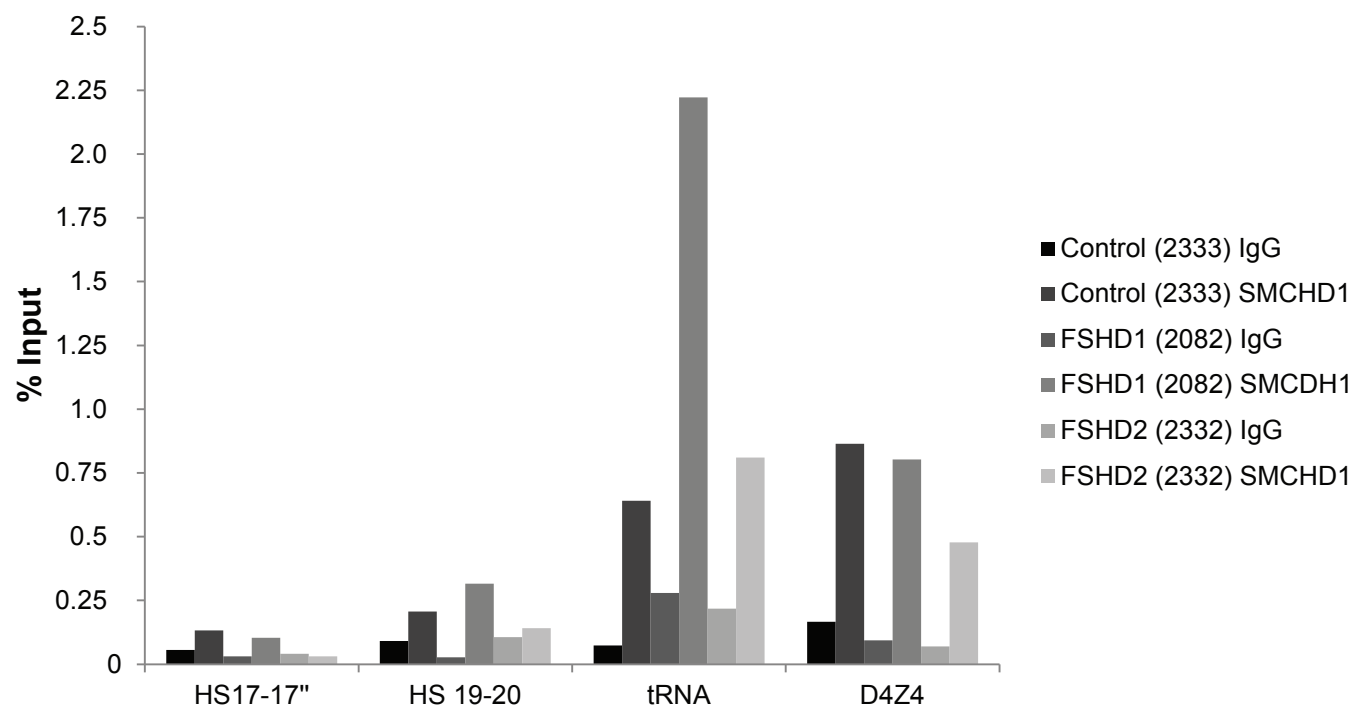

Supplement: Supplementary file 9 — Percent input ChIP-qPCR for representative individuals. ChIP-qPCR for all ChIP examined loci displayed as a percent of the input for one control, FSHD1, and FSHD2 individual. Please note that it is difficult to compare single copy loci, HS17-17′and HS19-20, to multi-copy loci tRNA and D4Z4. (PDF 831 kb) [file 13395_2017_129_MOESM9_ESM.pdf]
